# Socioeconomic factors associated with poor medication adherence in patients with type 2 diabetes

**DOI:** 10.1007/s00228-023-03571-8

**Published:** 2023-10-23

**Authors:** Marie Ekenberg, Miriam Qvarnström, Anders Sundström, Mats Martinell, Björn Wettermark

**Affiliations:** 1 Department of Pharmacy, Faculty of Pharmacy, Uppsala University, Uppsala, Sweden; 2 Department of Public Health and Caring Sciences, Uppsala University, Uppsala, Sweden

**Keywords:** Medication adherence, Persistence, Discontinuation, Diabetes mellitus, Type 2, Socioeconomic disparities in Health, Hypoglycemic agents

## Abstract

**Purpose:**

This study aims to determine initiation and persistence for patients with type 2 diabetes receiving their first prescription of an antidiabetic agent and the associations with socioeconomic factors.

**Methods:**

A cohort study including 8515 patients with type 2 diabetes who were prescribed their first antidiabetic medication between 2012 and 2019 in Uppsala, Sweden, was followed during 2 years. Medical records were linked to national registers on dispensed drugs and socioeconomic data. Adherence was assessed based on patients’ medication claims within 30 days of prescription (initiation) and continued claims after 24 months (persistence). Multivariable logistic regression was used to determine the associations with the socioeconomic factors age, sex, living status, country of birth, education, occupation, and income.

**Results:**

Within 30 days, 92.4% of the patients claimed their first prescription, and 64.0% were still being dispensed the initially prescribed medication after 24 months. Unemployed patients had lower initiation rates, and women had lower persistence rates. Factors associated with both low initiation and persistence were low income, young or old age, birth outside Europe, and being prescribed other diabetes drugs than metformin monotherapy.

**Conclusion:**

Socioeconomic factors have different impact on the initiation of a new medication and the persistence to treatment in type 2 diabetes. It is important to acknowledge these differences to develop appropriate interventions to improve medication nonadherence.

**Supplementary Information:**

The online version contains supplementary material available at 10.1007/s00228-023-03571-8.

## Introduction

Effective treatment of patients with type 2 diabetes mellitus (T2DM) includes lifestyle changes and medication to prevent morbidity and reduce costs [1–3]. However, several studies show that few patients achieve glycemic control in clinical practice, partly because of poor medication adherence (MA) [4–8]. The proportion of patients adherent to antidiabetic therapy varies between 33 and 93% after 6–24 months [9, 10]. MA can be divided into three different phases: (I) *initiation*, where treatment is started and the first dose administered; (II) *implementation*, which measure the extent to which the prescribed dosage regimen is followed; and (III) *persistence*, which represent the time from initiation to discontinuation of treatment [11]. Data sources have different validity depending on which phase of MA is assessed. Register data is considered to be the “gold standard,” the most truthful data source, in studies of measuring initiation and persistence [12]. In previous studies on T2DM patients, being middle aged, native persons, being prescribed other drugs than metformin, not having comorbidities, and high socioeconomic (SE) status are associated with higher MA [4, 13–16], while the results on sex differs between studies [13, 16]. The factors associated with poor MA differ depending on the aspect of the treatment period being assessed [17]. Factors associated with initiation are related to the consultation with the prescribing physician, reactions to diagnosis and/or medication, and patient beliefs and previous experiences. On the other hand, factors associated with persistence are more related to side effects and the lack of perceived treatment benefits [18].

There is a need for more research on MA applying clear definitions of adherence and separate assessment of initiation, implementation, or persistence and factors associated with each phase [15, 19]. Studies assessing determinants of MA in T2DM have found SE status to be associated with MA [13, 15, 16]. However, SE status is mainly based on income, or using a combined measure generating a deprivation score, and does not separate the different phases of MA. In this study, the term socioeconomic position (SEP) is used which is a broader term than SE status and also includes income, education, and occupation [20]. Knowledge about which individual socioeconomic characteristics that are associated with poor MA may be helpful in designing more individualized interventions to tackle medication nonadherence. This is important to ensure equitable healthcare, especially since patients with T2DM have lower SEP in general [21]. The initiation phase is rarely included in studies because of the lack of data on issued prescriptions that were never filled. Furthermore, studies that include all newly prescribed drugs are lacking, and few studies have longer follow-up than 12 months [4, 22]. In previous research, persistence has mainly been evaluated as persistence to the initially prescribed treatment. However, persistence to any antidiabetic therapy, including switching of antidiabetic agents, may be more clinically relevant. This study therefore aims to determine initiation and persistence for patients with T2DM receiving their first prescription of an antidiabetic agent and the associations with SE factors.

## Methods

### Population and data source

This cohort study included patients diagnosed with T2DM, without previous treatment for this condition, prescribed an antidiabetic agent between the 1st of January 2012 and to 31st of December 2019. Patients were eligible if over 18 years old at first prescription. All primary healthcare centers (PHC) in the region of Uppsala, Sweden, were asked to participate, and 48 out of 51 PHCs consented to participation. The study population was identified using ICD-10 code E11 in the electronic health record (EHR) for the region of Uppsala, and data were extracted on all patients’ primary and secondary care diagnoses, issued prescriptions, and laboratory data. This dataset was linked to two national registers; the Swedish Prescribed Drug Register provided data on drug name, ATC code, dispensing date, and SE data from Statistics Sweden including data on age, sex, country of birth, immigration, emigration, education, unemployment (days of unemployment per year or receiving unemployment aid), yearly income, receiving pension, and family status (registered partners/marriages and individuals in each household) [23, 24]. Data linkage was done by Statistics Sweden using personal identity numbers and pseudonymized before delivery of data [25].

### Variables

Antidiabetic agents were identified using the Anatomical Therapeutic Chemical classification system, ATC system [26]. The index date was defined as the date of the first issued prescription of an antidiabetic agent (ATC code A10) with no previous dispensing of any antidiabetic agent identified in the prescribed drug register, issued by any prescriber in Sweden within 24 months before the index date. Patients were considered exposed to treatment when dispensed an antidiabetic agent. Baseline data were collected 24 months before the index date and dispensing data 28 months after the index date. See Table S1 (Supplementary information) for a complete list of the ATC codes used.

Patient-related SE variables collected from Statistics Sweden were registered on the 31st of December every year. SE data from the previous year were used for patients who died or emigrated in the same year. Clinical variables extracted from EHR were defined as the last recorded value before the index date, but the covariate assessment window could expand to 24 months before the index date. Variables were grouped accordingly as follows: age groups: 18–49, 50–64, 65–79, and ≥ 80 years, due to previous found differences in MA between these age groups [13]; country of birth: Sweden, the rest of Europe, and the rest of the world; highest education: primary school (< 10 years), secondary school (10–12 years), and university (> 12 years); unemployment: long-term unemployment (≥ 183 days previous year) and other unemployment (short-term unemployment, part-time unemployment, or receiving unemployment benefits in the previous year). Individual income was calculated into quartiles. Family status was grouped accordingly as follows: married (married or registered partners living together), cohabiting (living with an adult, but not married or registered partners), and living alone (living without other adults). Estimated glomerular filtration rate (eGFR) was calculated in the EHR. P-creatinine and the revised Lund-Malmö GFR estimating equation were mostly used for calculating the eGFR in EHR [27]. eGFR was divided into levels of chronic kidney disease (CKD) scale: CKD 1 (GFR ≥ 90 ml/min/1.73m^2^), CKD 2 (GFR 60–89 ml/min/1.73m^2^), CKD 3a (GFR 45–59 ml/min/1.73m^2^), CKD 3b (GFR 30–44 ml/min/1.73m^2^), and CKD 4–5 (GFR < 30 ml/min/1.73m^2^) [28]. Glycosylated hemoglobin (HbA1c) was grouped as follows: ≤ 48, 49–69, and ≤ 70 mmol/mol. Diagnoses were defined using ICD-10 codes: hypertension (I10), depression (F32, F33, F34, F38, F39), obesity (E66), and cardiovascular diseases (ischemic heart diseases (I20–I25), peripheral vascular disease (I70–I79), stroke/transitional ischemic attack (TIA) (I63, G45.9), and heart failure (I50)). To be classified as having depression, patients could either have a diagnosis of depression or have claimed an antidepressant drug (ATC code N06A). Sex and family status were combined to determine possible sex differences in social support and MA. Four treatment groups were compared: (I) metformin monotherapy; (II) insulins, patients prescribed one or several insulins; (III) other monotherapy, monotherapy with any drug other than metformin or insulin; and (IV) polytherapy, patients prescribed either insulin in combination with other antidiabetic agents, several non-insulin antidiabetic agents, or fixed combination products (A10AE5 or A10BD).

This study assessed two aspects of MA, initiation and persistence [11]. Initiation was measured as the proportion of patients claiming their first prescribed antidiabetic agent at the pharmacy within 30 days from the prescription date (initiation of treatment, *I*_30_) and claiming two prescriptions within 150 days from the prescription date (initiation of long-term treatment, *I*_150_). Persistence refers to the period after initiation in which patients continue to take their medicine. Therefore, only patients who claimed their first prescription within *I*_30_ were included in the persistence analysis. Persistence (yes/no) was defined using the anniversary method: whether patients claim the antidiabetic agent within a specific time window [29]. Non-persistence was defined as no dispensed medication within 3 months before and after the endpoint 12 months (*P*_12_) and 24 months (*P*_24_) after the first dispensing date. The time window of ± 3 months was based on the Swedish reimbursement system, where patients normally receive a 3-month supply, including a grace period of 3 months (supply gap). Patients initiated on treatment may also receive a start package with up to 30 days of supply. To ensure coverage of medication costs, patients can obtain a refill once at least 2/3 of the previous supply is used [30]. A grace period of 3 months has previously been recommended to adjust for stockpiling [31]. Initiation and persistence were defined as claiming all the initially prescribed antidiabetic agents. If prescribed several medications, the mean number of days until they were dispensed was recorded.

### Statistical analysis

Descriptive statistics were used for baseline variables and patients defined as initiated on or persistent to therapy, respectively. Categorical data presents the proportions of patients, and continuous data presents mean values and standard deviations (SD). Covariate multicollinearity was tested with generalized variance inflation factor (GVIF) before using multivariable logistic regression models to estimate the effect of covariates on initiation and persistence. The full logistic regression model included all SE factors, and sensitivity analysis was done with reduced logistic regression models which included one SE factor in each model, both the full and reduced model adjust for age, CKD, HbA1c, comorbidities, prescribed treatment, and year of prescription. All results are presented as adjusted and crude odds ratios (OR) with confidence intervals of 95% (95% CI). All analyses for initiation were performed using the complete cohort of 8515 persons, and analysis for persistence was performed using the population initiated according to *I*_30_. Sensitivity analysis for initiation was performed with cumulative graphs of days until initiation, and sensitivity analysis for persistence was performed with a time window of ± 2 months. Data management was done in SAS Enterprise Guide 8.2, and analysis was done in RStudio version 2022.07.01 + 554.

## Results

A total of 12,962 patients with T2DM were prescribed an antidiabetic agent for the first time in the region of Uppsala between 2012 and 2019; 205 of them were excluded due to incomplete personal identification numbers, and 3885 patients were excluded due to dispensations of antidiabetics during the wash-out window, 311 persons due to immigration during the past 2 years, and further 46 patients due to missing data of all SE variables (Fig. S2**)**. The final population included 8515 patients, of which 77.2% were prescribed metformin monotherapy; the rest were prescribed either insulins (9.1%), other antidiabetic monotherapy (8.4%), or polytherapy with two or more antidiabetic agents (5.4%). Patients were initially prescribed one to four different agents. Other cohort baseline characteristics are presented in Table 1.

**Table 1:** Characteristics of patients with T2DM prescribed an antidiabetic agent for the first time 2012–2019. Clinical data were the latest recorded values before prescription, and socioeconomic data were collected 31st of December previous year. *SD*standard deviation

**Characteristics**	**Total population** ***N*(%)**		**Metformin monotherapy**		**Insulins**		**Other monotherapy**		**Polytherapy**	
**Demographic data**	**8515**	**(100)**	**6570**	**(77.2)**	**771**	**(9.1)**	**713**	**(8.4)**	**461**	**(5.4)**
**Age, mean, SD**	59.8, SD = 15.2		60.6, SD = 13.9		54.9, SD = 20.9		59.8, SD = 17.8		56.6, SD = 14.6	
18–49 years	1979	(23.2)	1329	(20.2)	298	(38.7)	202	(28.3)	150	(32.5)
50–64 years	2863	(33.6)	2307	(35.1)	173	(22.4)	216	(30.3)	167	(36.2)
65–79 years	3048	(35.8)	2542	(38.7)	200	(25.9)	182	(25.5)	124	(26.9)
≥ 80 years	625	(7.3)	392	(6.0)	100	(13.0)	113	(15.8)	20	(4.3)
**Sex and living status**										
**Men**	4699	(100.0)	3675	(55.9)	411	(53.3)	312	(43.8)	301	(65.3)
Living alone	1646	(35.0)	1202	(32.7)	186	(45.3)	124	(39.7)	134	(44.5)
Married/cohabiting	3053	(65.0)	2473	(67.3)	225	(54.7)	188	(60.3)	167	(55.5)
**Women**	3816	(100.0)	2895	(44.1)	360	(46.7)	401	(56.2)	160	(34.7)
Living alone	1509	(39.5)	1127	(38.9)	142	(39.4)	174	(24.8)	66	(41.3)
Married/cohabiting	2307	(60.5)	1768	(61.1)	218	(60.6)	227	(75.2)	94	(58.7)
**Country of birth**										
Sweden	6599	(77.5)	5107	(77.7)	589	(76.4)	585	(82.0)	318	(69.0)
Other European countries	750	(8.8)	570	(8.7)	73	(9.5)	59	(8.3)	48	(10.4)
Rest of the world	1166	(13.7)	893	(13.6)	109	(14.1)	69	(9.7)	95	(20.6)
**Educational level**										
Primary	2246	(26.4)	1719	(26.2)	203	(26.3)	192	(26.9)	132	(28.6)
Secondary	3781	(44.4)	2955	(45.0)	313	(40.6)	314	(44.0)	199	(43.2)
University	2381	(28.0)	1831	(27.9)	241	(31.3)	197	(27.6)	112	(24.3)
Missing	107	(1.3)	65	(1.0)	14	(1.8)	10	(1.4)	18	(3.9)
**Occupation**										
Employed/working	3802	(44.7)	2882	(43.9)	369	(47.9)	321	(45.0)	230	(49.9)
Retired	4179	(49.1)	3309	(50.4)	344	(44.6)	354	(49.6)	172	(37.3)
Long-term unemployment	154	(1.8)	117	(1.8)	10	(1.3)	7	(1.0)	20	(4.3)
Other unemployment	380	(4.5)	262	(4.0)	48	(6.2)	31	(4.3)	39	(8.5)
**Income**										
1st quartile	2128	(25)	1472	(22.4)	282	(36.6)	218	(30.6)	156	(33.8)
2nd quartile	2127	(25)	1626	(24.7)	211	(27.4)	203	(28.5)	87	(18.9)
3rd quartile	2131	(25)	1692	(25.8)	175	(22.7)	161	(22.6)	103	(22.3)
4th quartile	2129	(25)	1780	(27.1)	103	(13.4)	131	(18.4)	115	(24.9)
**Health data**										
**GFR, mean, SD**	75.1, SD = 16.0		76.3, SD = 13.0		70.9, SD = 25.0		66.0, SD = 22.9		80.0, SD = 15.0	
CKD1	1536	(18.0)	1047	(15.9)	221	(28.7)	129	(18.1)	139	(30.2)
CKD2	3382	(39.7)	2864	(43.6)	172	(22.3)	206	(28.9)	140	(30.4)
CKD3a	613	(7.2)	443	(6.7)	57	(7.4)	87	(12.2)	26	(5.6)
CKD3b	184	(2.2)	59	(0.9)	44	(5.7)	75	(10.5)	6	(1.3)
CKD4, CKD5	101	(1.2)	5	(0.1)	51	(6.6)	41	(5.8)	4	(0.9)
Missing	2699	(31.7)	2152	(32.8)	226	(29.3)	175	(24.5)	146	(31.7)
**HbA1c, mean, SD**	60.2, SD = 22.0		57.2, SD = 17.6		78.9, SD = 32.2		50.6, SD = 19.0		93.2, SD = 26.0	
≤ 48 mmol/mol	2288	(26.9)	1839	(28.0)	130	(16.9)	298	(41.8)	21	(4.6)
49–69 mmol/mol	3039	(35.7)	2702	(41.1)	106	(13.7)	186	(26.1)	45	(9.8)
≥ 70 mmol/mol	1590	(18.7)	935	(14.2)	300	(38.9)	67	(9.4)	288	(62.5)
Missing	1598	(18.8)	1094	(16.7)	235	(30.5)	162	(22.7)	107	(23.2)
**Comorbidities**										
Hypertension	4004	(47.0)	3289	(50.1)	238	(30.9)	303	(42.5)	174	(37.7)
Cardiovascular^a^	1182	(13.9)	860	(13.1)	111	(14.4)	146	(20.5)	65	(14.1)
Depression	1589	(18.7)	1171	(17.8)	123	(16.0)	226	(31.7)	69	(15.0)
Obesity	966	(11.3)	621	(9.5)	32	(4.2)	299	(37.3)	47	(10.2)

*SD*standard deviation

^a^Cardiovascular disease groups include ischemic heart diseases (I20–I25), peripheral vascular disease (I70–I79), stroke/TIA (I63, G45.9), and heart failure (I50)

**Fig. 1:**
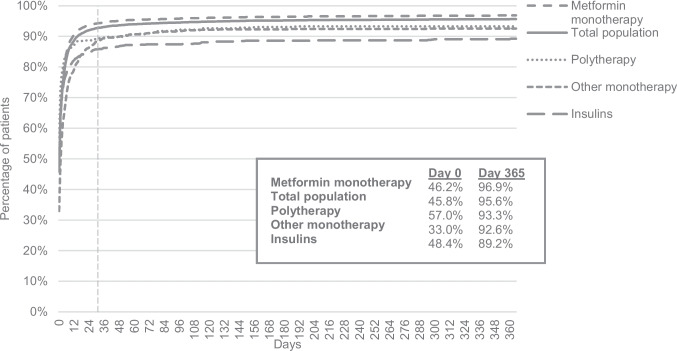
Cumulative graph of days until the first dispensation of an antidiabetic medication for the total population and each treatment group (metformin monotherapy, insulins, other antidiabetic monotherapy, or polytherapy)

**Fig. 2:**
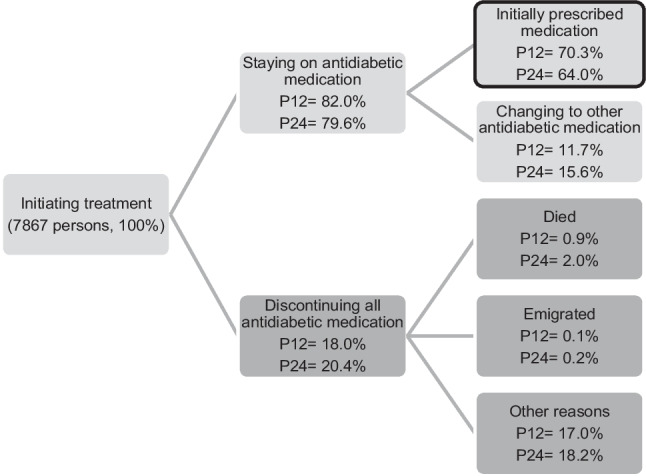
Percentages of patients staying on antidiabetic medications (persistence to any antidiabetic medications, upper branch), staying on initially prescribed antidiabetic medications (circled box), and patients discontinuing (lower branch) at 12 (*P*_12_) and 24 months (*P*_24_) after first dispensation date

Most patients claimed their prescription soon after it was issued: 84.8% within the first 7 days and 92.4% within a month (*I*_30_) (Fig. 1). Only 3.7% never claimed their antidiabetic medications within 365 days (last valid date of prescriptions). The number of days until the second dispensation varied more; 71.4% claimed it within 150 days (*I*_150_), and 16.3% of patients did not claim the second dispensation within 365 days. Persistence was calculated for the population initiating treatment at *I*_30_, 7867 persons (92.4% of the study population). There were 70.3% who claimed all their initially prescribed agents within the 12 months ± 3 months from dispensing date (*P*_12_) and 64.1% within *P*_24_ (Fig. 2).

**Fig. 3:**
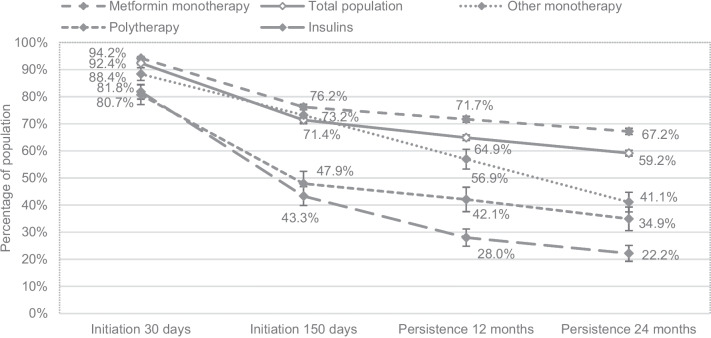
Percentage of patients with 95% confidence intervals in each treatment group claiming the first prescription from a pharmacy (first dispensing date) within 30 days from prescription date (*I*_30_), claiming the second prescription within 150 days from prescription date (*I*_150_), claiming the prescription in the time window 12 ± 3 months after the first dispensing date (*P*_12_*), and claiming the prescription in the time window 24 ± 3 months after the first dispensing date (*P*_24_*). *This figure shows percentages of all patients initially prescribed that specific medication and not only persistence for patient initiating (*I*_30_); therefore, all calculations in this figure are based on the full study population of 8515 persons

There were differences in initiation and persistence depending on the type of prescribed medication (Fig. 3). Ninety-four percent of the patients prescribed metformin were initiated at *I*_30_, compared to 80% for insulin and polytherapy. At *P*_24_, 67% of the patients prescribed metformin were persistent, compared to 22% for insulins. Significant differences compared to metformin were observed for all other medication regimens, except for the group with other monotherapy in *I*_150_. Logistic regression analysis showed that patients prescribed metformin monotherapy had significantly higher initiation at *I*_30_ and higher persistence at *P*_12_ and *P*_24_, compared to all other treatments. For *I*_150_, initiation was significantly higher compared to insulins and other antidiabetic monotherapy.

**Fig. 4:**
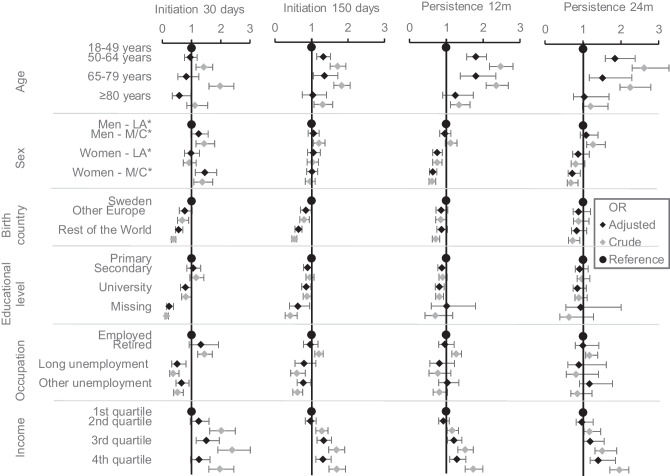
Forest plot including adjusted^α^ and crude odds ratios (OR) with 95% confidence intervals for factors associated with initiation of treatment before 30 days (*I*_30_), initiation of second dispensation before 150 days (*I*_150_), persistence with treatment after 12 months (*P*_12_), and persistence with treatment after 24 months (*P*_24_). *Abbreviations: LA, living alone; M/C, married/cohabiting. ^α^Values were adjusted for all other variables in the figure and, additionally, prescribed treatment (metformin monotherapy, insulins, other antidiabetic monotherapy, and polytherapy), CKD level, HbA1c, hypertension, cardiovascular disease, depression, obesity, and year of prescription

There were some differences between the factors associated with initiation and persistence. The SE groups with lowest initiation were unemployed or persons born outside Europe (*I*_30_: 80–85%; I_150_: 58–60%). The SE groups with the lowest persistence were young, unemployed, or persons born outside Europe (*P*_12_: 50–54%; P_24_: 44–50%). For percentages for all the groups, see Table S3. Combining different SE factors was restricted due to small samples in each group, resulting in large confidence intervals, but of the young women born outside of Sweden, only 30% were persistent after 24 months (see Fig. S4). In regression analysis, being unemployed was only associated with lower initiation, while being female was only associated with lower persistence. Factors associated with both lower initiation and persistence were being born outside Europe and low income. Being under 50 years old, over 80 years, or having a university education was associated with lower *I*_150_, *P*_12_, and *P*_24_ but not *I*_30_ (Fig. 4). All factors remained significant when using a 99% confidence interval except for *I*_30_: other unemployment; *I*_150_: age 65–79, other Europe, university education, other employment; and *P*_12_: income for the 3rd quartile (Table S5).

Another regression analysis estimated the associations of the same factors and patients persistent to antidiabetic therapy, instead of persistence to the initially prescribed antidiabetic agents. At 24 months, approximately 80% of patients continued with any antidiabetic medication, while 64% remained persistent to the initially prescribed agents (Fig. 2). Logistic regression analysis performed for factors associated with persistence to any antidiabetic therapy corresponded to factors associated with persistence to initially prescribed agents in Fig. 4; however, patients born outside Europe had a stronger association of not being persistent, and patients with a university education did not have significantly lower persistence than the reference group (Table S6).

There were 309 individuals in the population who died within the study period of 28 months. Of these, 56.2% were defined as persistent at *P*_12_ and 44.9% at *P*_24_. Only 24 persons emigrated within the total study period of 28 months. When logistic regression analysis was performed after excluding patients who had emigrated or died, the results were very similar. The associations for age groups did not change nor did the associations for birth country. Other sensitivity analysis was performed based on different definitions of initiation (*I*_30_ and *I*_150_) and persistence (*P*_12_ and *P*_24_). Cumulative graphs were used for initiation. The plateau at day 25 (92% dispensed), shown in Fig. 1, supported the decision of a 30-day cut-off for *I*_30_. For *I*_150_, the plateau occurs close to the mean value of 144 days until the second dispensation, which in addition to the predicted supply supports a 150-day cut-off. Sensitivity analysis for persistence was performed with a time window of 12 ± 2 months and 24 ± 2 months instead of ± 3 months (Table S7). Subsequently, 63.9% were defined as persistent for *P*_12_ and 57.9% for *P*_24_. Reduced logistic regression models adjusting for one SE factor in each model found similar results as shown in Fig. 4, but larger effects for birth country, income, and occupation which are more similar to the crude models.

## Discussion

In this study on 8515 primary care patients receiving their first prescription of an antidiabetic drug, the majority claimed their prescription. However, many of them never claimed a second prescription, and 64% remained on the initially prescribed drug 2 years later. Patients who failed to claim their prescription within 30 days were more likely to be born outside of Europe or unemployed. Factors associated with the 30% of patients not starting long-term treatment (i.e., not claiming the second prescription) included being born outside of Europe, unemployment, low income, and being either young or old. Low income, age, and birth outside Europe continued to impact the use of antidiabetic medications for persistence, and the factors associated with discontinuation also included females and having a university education.

We found that 92% of patients claimed their prescription within 30 days from the prescription date. This initiation rate aligns with previous research, which suggests that, on average, 10% of patients never claim their diabetes prescriptions [32]. However, in our study, most patients claimed their first prescription within a year. Therefore, using time windows that are too short may underestimate the initiation. Canadian researchers found one-third to discontinue within 3 months, being similar to the findings of 71% for *I*_150_ [14]. In our study, the pattern of initiation was similar across different treatment groups, but patients prescribed only insulins were more likely to never claim their prescription. Early discontinuation of insulins could be explained by some patients using them temporarily for high HbA1c levels at the time of prescription, while another antidiabetic agent is prescribed for long-term use. Polytherapy was also discontinued early, potentially for the same reason as for insulins. Although other studies have shown that insulins have lower MA compared to other antidiabetic therapies, our findings support this observation [19]. Patients prescribed monotherapies other than metformin or insulins were less likely to claim their prescriptions. This could be due to the relatively higher cost of the new antidiabetic agents compared to metformin and insulins. Additionally, insulin is fully reimbursed in Sweden without any patient co-payment. Metformin users had higher frequencies of both initiation and persistence, but still, 32.8% of patients had discontinued metformin after 24 months, and the other treatments had even higher discontinuation rates. Overall, 70% of the total population demonstrated persistence after 1 year, and 64% remained persistent after 2 years. These rates are slightly higher than previous research, which reported persistence rates for initially prescribed antidiabetic agents ranging between 33 and 61% after 6–24 months [9]. The generous definition of persistence with the anniversary method, using a time window of ± 3 months, contributed to higher rates of persistence in our study. Differences between studies may also be attributed to variations in study design or the populations included.

Demographic factors, such as age and sex, were identified as being associated with MA. The oldest age group exhibited lower treatment initiation rates, potentially due to practical challenges related to collecting the medication or the presence of comorbidities. However, both the youngest and the oldest age groups demonstrated lower persistence rates. Previous studies have shown associations between poor MA and either older age [16] or both very young and old age [13]. Patients between 50 and 79 years may be more motivated by the potential for a longer life. Women had lower persistence compared to men, which contradicts some research findings [13, 33] but aligns with other studies [16, 34]. Sex patterns were observed for both persistence to the initially prescribed medication and overall antidiabetic therapy. This could be attributed to women having higher adherence to lifestyle interventions involving diet and physical activity, thus reducing the need for medication. Discontinuing women had lower initial HbA1c levels, with a mean of 54.9 mmol/mol, compared to the total population mean of 60.2 mmol/mol. Unemployment and being born outside of Europe were the primary SE factors associated with low initiation. However, unemployment was not associated with persistence, indicating a temporary effect on adherence, potentially due to changes in routines or increased stress. This could also be influenced by the Swedish reimbursement system, due to higher patient co-payment for initial dispensations. Patients born outside Europe exhibited lower adherence across all measures, aligning with studies where non-native background was a disadvantage for adherence [15, 34, 35]. Low income was associated with lower initiation and persistence, which corresponds to the association between low SE status and poor MA reported in other studies [34]. University education was slightly associated with lower persistence, but this was not significant with a 99% confidence interval. While being married has been associated with adherence in other studies [6, 13], we only observed a slightly higher degree of claiming the first prescription among married individuals. No associations were found when comparing patients living alone to those who were married or cohabiting or when separating the married and cohabiting groups. It was not possible to stratify many SE factors in the analysis due to small samples in each group, but a pattern was seen where young women born outside of Sweden had very low persistence, which calls for further research.

### Strengths and limitations

This study has several notable strengths. Firstly, the separation of initiation and persistence phases and the analysis of associated factors separately provide valuable insights into targeting specific adherence phases. The findings of this study demonstrate that different factors play a significant role during different phases of adherence. Secondly, the study assessed two distinct measures for initiation, distinguishing between patients who tried the medication and those who started long-term treatment. The comparison of EHR prescriptions with pharmacy claims for initiation is an understudied aspect in MA research, offering a better understanding of patients’ therapy initiation. Additionally, the study’s 2-year follow-up period after the first dispensation date allows for tracking patients’ prescription renewal in the second year. The use of national dispensing data is also advantageous, as it encompasses all dispensed drugs in Sweden, ensuring comprehensive coverage for all patients and the ability to track patients who relocate within the country. Lastly, the availability of individual-level data on various SE factors is a rarity in the field, contributing new insights.

While the national Swedish registers provide extensive data coverage, certain limitations exist. For instance, approximately 1.3% of the study sample had missing data on education, primarily due to patients not being born in Sweden [24]. Clinical variables and diagnoses, although highly covered in EHR, possess some limitations due to their lack of structure compared to national registers. Missing data may occur in EHR variables due to the data collection time frame. The calculated eGFR was missing for 31% of patients, and 19% did not have any HbA1c measurements within 12 months before prescription. This could be attributed to HbA1c test being conducted before prescription of the antidiabetic drug and added to the EHR when analyzed at the laboratory, a few days later. Another limitation is the inability to adjust for other health-related factors such as body mass index (BMI) or smoking due to missing data, which may result in residual confounding. Income was only based on individual income and not household income, which would be more accurate when determining SE position. Another limitation for income is using the same data collection window as for the other variables, where a mean of several years might reflect SE position better. The main limitation of the regression model is the general correlation between different SE factors. Although multicollinearity testing only showed high correlations between age and occupation, in the reduced models adjusting for each SE factor separately, the effects of high income, unemployment, and country of birth were more pronounced. This suggests some overfitting of the full model and underestimation of the impact of these factors on initiation and persistence. There may also be clustering in data with patients visiting the same doctor or PHC, which could have been incorporated in a multilevel analysis. Furthermore, there are also some limitations in the calculations of persistence. The anniversary method is a rough estimation of a person’s actual usage and likely overestimates persistence. Other methods including supply gaps could, on the contrary, underestimate persistence, due to challenges in estimating stockpiling and undocumented therapy changes. Finally, when performing many statistical tests, there is always the risk of by chance gaining associations. We addressed it by adding 99% confidence intervals for the SE variables, which showed similar results.

The Uppsala region included in this study is representative of the entire country of Sweden, including both urban and rural areas and both low- and high-income neighborhoods. The generalizability of this study beyond Sweden is limited due to differences in regulations and laws for prescribing, dispensing, and reimbursement between countries.

In conclusion, the findings of this study reveal that while a majority of patients claim their prescriptions, certain SE groups exhibit lower rates of initiation or persistence. To address medication nonadherence effectively, patients from the different SE groups need support in different phases of MA. Patients born outside of Europe or with a low income might need support for both the initiation and persistence phase, while unemployed might need more support for the initiation phase and patients with young or old age for the persistence phase. By supporting patients in the phase of need, it is possible to improve MA and reduce inequalities in quality of care.

### Supplementary Information

Below is the link to the electronic supplementary material.

Supplementary file1 (PDF 42 KB)

Supplementary file2 (PDF 61 KB)

Supplementary file3 (PDF 135 KB)

Supplementary file4 (PDF 120 KB)

Supplementary file5 (PDF 235 KB)

Supplementary file6 (PDF 116 KB)

Supplementary file7 (PDF 169 KB)

## Data Availability

The pseudonymized patient-level data collected from the electronic health records and national registers are not allowed to share publicly due to confidentiality reasons; however, upon reasonable request, additional analyses can be conducted after contact with the corresponding author.

## References

[1] Davies MJ, Aroda VR, Collins BS, Gabbay RA, Green J, Maruthur NM et al (2022) Management of hyperglycemia in type 2 diabetes. A consensus report by the American Diabetes Association (ADA) and the European Association for the Study of Diabetes (EASD). Diabetes Care 28;45(11):2753–2786. 10.2337/dci22-0034 10.2337/dci22-0034 PMC10008140 36148880

[2] Sun H, Saeedi P, Karuranga S, Pinkepank M, Ogurtsova K, Duncan BB et al (2022) IDF Diabetes Atlas: global, regional and country-level diabetes prevalence estimates for 2021 and projections for 2045. Diabetes Res Clin Pract 183:109119. 10.1016/j.diabres.2021.109119 10.1016/j.diabres.2021.109119 PMC11057359 34879977

[3] Hellgren M, Svensson A, Franzén S, Ericsson Å, Gudbjörnsdottir S, Ekström N et al (2021) The burden of poor glycaemic control in people with newly diagnosed type 2 diabetes in Sweden: a health economic modelling analysis based on nationwide data. Diabetes Obes Metab 23(7):1604–1613. 10.1111/dom.14376 10.1111/dom.14376 PMC8360155 33729661

[4] Tang Y, Weiss T, Liu J, Rajpathak S, Khunti K (2020) Metformin adherence and discontinuation among patients with type 2 diabetes: a retrospective cohort study. J Clin Transl Endocrinol 1 20:100225. 10.1016/j.jcte.2020.100225 10.1016/j.jcte.2020.100225 PMC7240214 32461914

[5] Aikens JE, Piette JD (2013) Longitudinal association between medication adherence and glycaemic control in type 2 diabetes. Diabet Med 30(3):338–344. 10.1111/dme.12046 10.1111/dme.12046 PMC3567301 23075262

[6] Raum E, Krämer HU, Rüter G, Rothenbacher D, Rosemann T, Szecsenyi J et al (2012) Medication non-adherence and poor glycaemic control in patients with type 2 diabetes mellitus. Diabetes Res Clin Pract 1 97(3):377–384. 10.1016/j.diabres.2012.05.026 10.1016/j.diabres.2012.05.026 22763108

[7] Guerci B, Chanan N, Kaur S, Jasso-Mosqueda JG, Lew E (2019) Lack of treatment persistence and treatment nonadherence as barriers to glycaemic control in patients with type 2 diabetes. Diabetes Ther 10(2):437–449. 10.1007/s13300-019-0590-x 10.1007/s13300-019-0590-x PMC6437240 30850934

[8] Evans M, Engberg S, Faurby M, Fernandes JDDR, Hudson P, Polonsky W (2022) Adherence to and persistence with antidiabetic medications and associations with clinical and economic outcomes in people with type 2 diabetes mellitus: a systematic literature review. Diabetes Obes Metab 1;24(3):377–390. 10.1111/dom.14603 10.1111/dom.14603 PMC9299643 34779107

[9] Iglay K, Cartier SE, Rosen VM, Zarotsky V, Rajpathak SN et al (2015) Meta-analysis of studies examining medication adherence, persistence, and discontinuation of oral antihyperglycemic agents in type 2 diabetes. Curr Med Res Opin 31(7). 10.1185/03007995.2015.1053048 10.1185/03007995.2015.1053048 26023805

[10] Krass I, Schieback P, Dhippayom T (2015) Adherence to diabetes medication: a systematic review. Diabet Med 32(6):725–737. 10.1111/dme.12651 10.1111/dme.12651 25440507

[11] Vrijens B, De Geest S, Hughes DA, Przemyslaw K, Demonceau J, Ruppar T et al (2012) A new taxonomy for describing and defining adherence to medications. Br J Clin Pharmacol 73(5). 10.1111/j.1365-2125.2012.04167.x 10.1111/j.1365-2125.2012.04167.x PMC3403197 22486599

[12] Elseviers M, Wettermark B, Almarsdottir AB, Anderssen M, Benko R et al (2016) Drug utilization research: methods and applications. 1^st^ edn. John Wiley & Sons, Incorporated, Hoboken, pp 367

[13] Kardas P, Lewek P, Matyjaszczyk M (2013) Determinants of patient adherence: a review of systematic reviews. Front Pharmacol 4:91. 10.3389/fphar.2013.00091 10.3389/fphar.2013.00091 PMC3722478 23898295

[14] Campbell DJT, Campbell DB, Ogundeji Y, Au F, Beall R, Ronksley PE et al (2021) First-line pharmacotherapy for incident type 2 diabetes: prescription patterns, adherence and associated costs. Diabet Med 38(9):1–11. 10.1111/dme.14622 10.1111/dme.14622 34133781

[15] Capoccia K, Odegard PS, Letassy N (2016) Medication adherence with diabetes medication: a systematic review of the literature. Diabetes Educ 42(1). 10.1177/0145721715619038 10.1177/0145721715619038 26637240

[16] Kirkman MS, Rowan-Martin MT, Levin R, Fonseca VA, Schmittdiel JA, Herman WH et al (2015) Determinants of adherence to diabetes medications: findings from a large pharmacy claims database. Diabetes Care 38(4):604–609. 10.2337/dc14-2098 10.2337/dc14-2098 PMC4370331 25573883

[17] Maffoni M, Traversoni S, Costa E, Midão L, Kardas P et al (2020) Medication adherence in the older adults with chronic multimorbidity: a systematic review of qualitative studies on patient’s experience. Eur Geriatr Med 11(3):369–381. 10.1007/s41999-020-00313-2 10.1007/s41999-020-00313-2 32297271

[18] Cassimatis M, Kavanagh DJ, Smith AC (2014) Perceived needs for supported self-management of type 2 diabetes: a qualitative investigation of the potential for a web-based intervention. Aust Psychol 49(2):75–85. 10.1111/ap.12050

[19] McGovern A, Tippu Z, Hinton W, Munro N, Whyte M, de Lusignan S (2018) Comparison of medication adherence and persistence in type 2 diabetes: a systematic review and meta-analysis. Diabetes Obes Metab 20(4). 10.1111/dom.13160 10.1111/dom.13160 29135080

[20] Krieger N, Williams DR, Moss NE (1997) Measuring social class in US public health research: concepts, methodologies, and guidelines. Annu Rev Public Health 18(1):341–378. 10.1146/annurev.publhealth.18.1.341 10.1146/annurev.publhealth.18.1.341 9143723

[21] Wemrell M, Bennet L, Merlo J (2019) Understanding the complexity of socioeconomic disparities in type 2 diabetes risk: a study of 4.3 million people in Sweden. BMJ Open Diabetes Res Care 7(1):e000749. 10.1136/bmjdrc-2019-000749 10.1136/bmjdrc-2019-000749 PMC6861116 31798898

[22] Khunti K, Gomes MB, Kosiborod M, Nicolucci A, Pocock S, Rathmann W et al (2020) Metformin discontinuation in patients beginning second-line glucose-lowering therapy: results from the global observational DISCOVER study programme. BMJ Open 10(8). 10.1136/bmjopen-2019-034613 10.1136/bmjopen-2019-034613 PMC7462233 32868349

[23] Wettermark B, Hammar N, Fored CM, Leimanis A, Otterblad Olausson P, Bergman U et al (2007) The new Swedish Prescribed Drug Register--opportunities for pharmacoepidemiological research and experience from the first six months. Pharmacoepidemiol Drug Saf 16(7):726–735. 10.1002/pds.1294 10.1002/pds.1294 16897791

[24] Ludvigsson JF, Svedberg P, Olén O, Bruze G, Neovius M (2019) The longitudinal integrated database for health insurance and labour market studies (LISA) and its use in medical research. Eur J Epidemiol 34(4):423–437. 10.1007/s10654-019-00511-8/10.1007/s10654-019-00511-8 PMC6451717 30929112

[25] Ludvigsson JF, Otterblad-Olausson P, Pettersson BU, Ekbom A (2009) The Swedish personal identity number: possibilities and pitfalls in healthcare and medical research. Eur J Epidemiol 24(11):659–667. 10.1007/s10654-009-9350-y 10.1007/s10654-009-9350-y PMC2773709 19504049

[26] WHOCC - ATC/DDD Index [Internet]. [cited 2022 Jun 13]. https://www.whocc.no/atc_ddd_index/?code=A10B&showdescription=yes

[27] Nyman U, Grubb A, Larsson A, Hansson LO et al (2014) The revised Lund-Malmö GFR estimating equation outperforms MDRD and CKD-EPI across GFR, age and BMI intervals in a large Swedish population. Clin Chem Lab Med 52(6):815–824. 10.1515/cclm-2013-0741 10.1515/cclm-2013-0741 24334413

[28] National Kidney Foundation (2002). K/DOQI clinical practice guidelines for chronic kidney disease: evaluation, classification, and stratification. Am J Kidney Dis Off J Natl Kidney Found.

[29] Caetano PA, Lam JMC, Morgan SG (2006) Toward a standard definition and measurement of persistence with drug therapy: examples from research on statin and antihypertensive utilization. Clin Ther 28(9):1411–1424; discussion 1410. 10.1016/j.clinthera.2006.09.021 10.1016/j.clinthera.2006.09.021 17062314

[30] Så fungerar högkostnadsskyddet - Tandvårds- och läkemedelsförmånsverket TLV [Internet]. [cited 2023 Mar 16]. https://www.tlv.se/lakemedelsforetag/hogkostnadsskyddet/sa-fungerar-hogkostnadsskyddet.html

[31] Parker MM, Moffet HH, Adams A, Karter AJ (2015) An algorithm to identify medication nonpersistence using electronic pharmacy databases. J Am Med Inform Assoc JAMIA 22(5):957–961. 10.1093/jamia/ocv054 10.1093/jamia/ocv054 PMC5009927 26078413

[32] Cheen MHH, Tan YZ, Oh LF, Wee HL, Thumboo J (2019) Prevalence of and factors associated with primary medication non-adherence in chronic disease: a systematic review and meta-analysis. Int J Clin Pract 73(6):e13350. 10.1111/ijcp.13350 10.1111/ijcp.13350 30941854

[33] Jankowska-Polańska B, Świątoniowska-Lonc N, Karniej P, Polański J, Tański W, Grochans E (2021) Influential factors in adherence to the therapeutic regime in patients with type 2 diabetes and hypertension. Diabetes Res Clin Pract 173:108693. 10.1016/j.diabres.2021.108693 10.1016/j.diabres.2021.108693 33592212

[34] Rolnick SJ, Pawloski PA, Hedblom BD, Asche SE, Bruzek RJ (2013) Patient characteristics associated with medication adherence. Clin Med Res 11(2):54–65. 10.3121/cmr.2013.1113 10.3121/cmr.2013.1113 PMC3692389 23580788

[35] Mayberry LS, Bergner EM, Chakkalakal RJ, Elasy TA, Osborn CY (2016) Self-care disparities among adults with type 2 diabetes in the USA. Curr Diab Rep 16(11):113. 10.1007/s11892-016-0796-5 10.1007/s11892-016-0796-5 PMC5096842 27671320

