# Supplementary material for: Socioeconomic factors associated with poor medication adherence in patients with type 2 diabetes
Source: Eur J Clin Pharmacol. 2023 Oct 23;80(1):53–63. doi: 10.1007/s00228-023-03571-8 (PMC10781833; doi:10.1007/s00228-023-03571-8)
Supplement: Supplementary file 1 — Supplementary file1 (PDF 42 KB) [file 228_2023_3571_MOESM1_ESM.pdf]

Socioeconomic factors associated with poor medication adherence for patients with type 2 diabetes

Marie Ekenberg[1], Miriam Qvarnström[1], Anders Sundström[1], Mats Martinell[2], Björn Wettermark[1]

1 Department of Pharmacy, Faculty of Pharmacy, Uppsala University, Uppsala, Sweden. *marie.ekenberg@farmaci.uu.se*.

2 Department of Public Health and Caring Sciences, Uppsala University, Uppsala, Sweden.

Supplementary Table S1 Antidiabetic agents and ATC codes included in the dataset of prescribed medications.

Antidiabetic agents	ATC codes
**Insulin and analogues**	**A10A**
Insulins and analogues for injection, fast-acting	A10AB
Insulins and analogues for injection, intermediate-acting	A10AC
Insulins and analogues for injection, intermediate- or long-acting combined with fast-acting	A10AD
Insulins and analogues for injection, long-acting	A10AE
**Blood glucose lowering agents excluding Insulin**	**A10B**
**Biguanide derivatives**	**A10BA**
Metformin	A10BA02
**Sulfonylurea compounds**	**A10BB**
Glibenclamide	A10BB01
Glipizide	A10BB07
Glimepiride	A10BB12
**Peroral diabetes drugs, combinations**	**A10BD**
metformin and sitagliptin	A10BD07
metformin and vildagliptin	A10BD08
metformin and linagliptin	A10BD11
metformin and empagliflozin	A10BD20
metformin and ertugliflozin	A10BD23
**Glycosidase inhibitors**	**A10BF**
Acarbose	A10BF01
**Thiazolidinediones**	**A10BG**
Pioglitazone	A10BG03
**Dipeptidyl peptidase-4 (DPP-4) inhibitors**	**A10BH**
Sitagliptin	A10BH01
Saxagliptin	A10BH03
Linagliptin	A10BH05
**Glucagon-like peptide-1 (GLP-1) analogues**	**A10BJ**
Liraglutid	A10BX07 (before 2017), A10BJ02 (after 2017)
Dulaglutid	A10BJ05
Semaglutide	A10BJ06
**Sodium-glucose co-transporter 2 (SGLT2) inhibitors**	**A10BK**
Dapagliflozin	A10BX09 (before 2017), A10BK01 (after 2017)
Empagliflozin	A10BK03
**Other blood glucose lowering agents excluding Insulin**	**A10BX**
Repaglinide	A10BX02
