# Supplementary material for: Socioeconomic factors associated with poor medication adherence in patients with type 2 diabetes
Source: Eur J Clin Pharmacol. 2023 Oct 23;80(1):53–63. doi: 10.1007/s00228-023-03571-8 (PMC10781833; doi:10.1007/s00228-023-03571-8)
Supplement: Supplementary file 2 — Supplementary file2 (PDF 61 KB) [file 228_2023_3571_MOESM2_ESM.pdf]

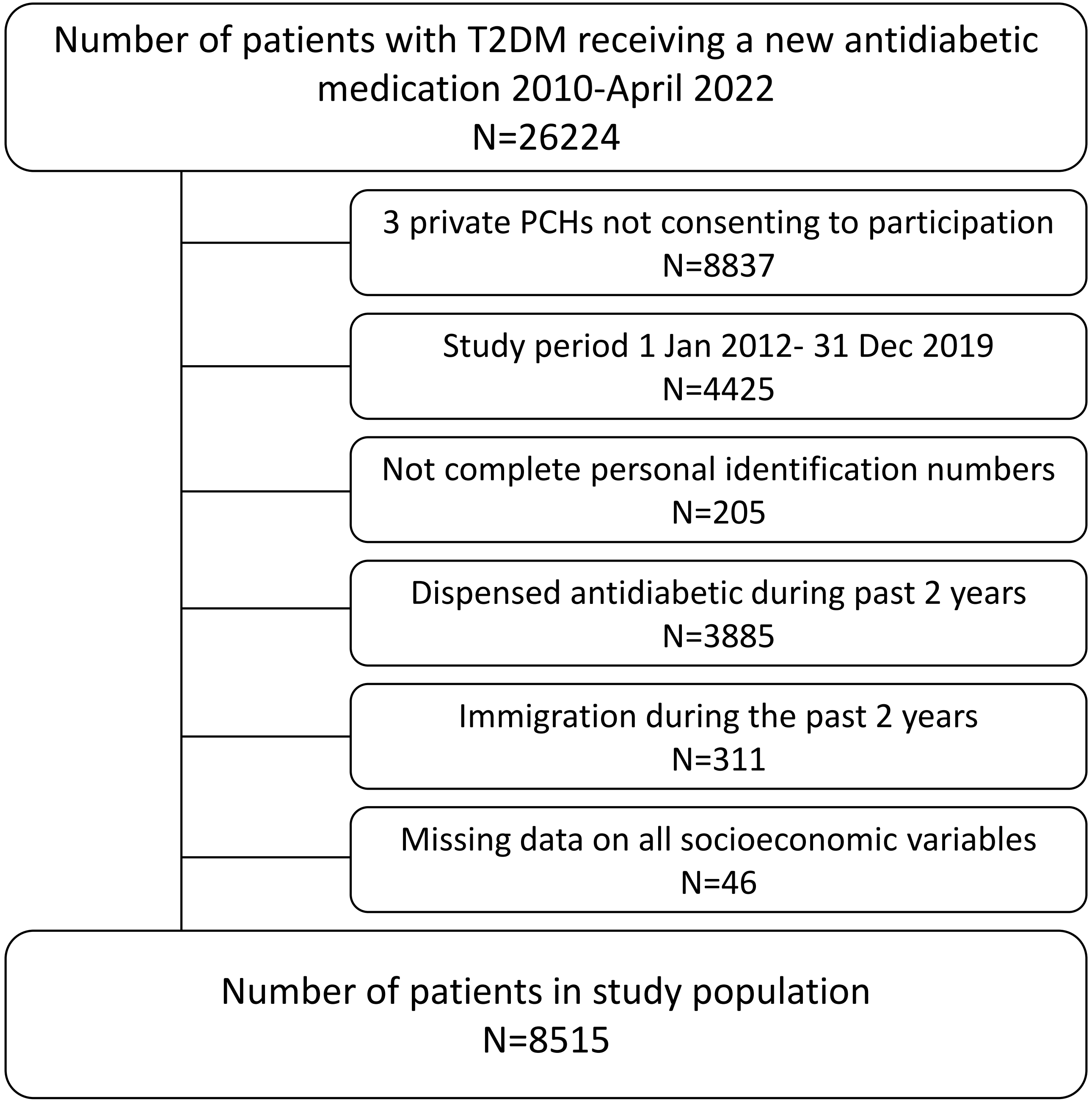
Number of patients with T2DM receiving a new antidiabetic medication 2010-April 2022
N=26224
3 private PCHs not consenting to participation
N=8837
Study period 1 Jan 2012- 31 Dec 2019
N=4425
Not complete personal identification numbers
N=205
Dispensed antidiabetic during past 2 years
N=3885
Immigration during the past 2 years
N=311
Missing data on all socioeconomic variables
N=46
Number of patients in study population
N=8515
