# Supplementary material for: Socioeconomic factors associated with poor medication adherence in patients with type 2 diabetes
Source: Eur J Clin Pharmacol. 2023 Oct 23;80(1):53–63. doi: 10.1007/s00228-023-03571-8 (PMC10781833; doi:10.1007/s00228-023-03571-8)
Supplement: Supplementary file 3 — Supplementary file3 (PDF 135 KB) [file 228_2023_3571_MOESM3_ESM.pdf]

Socioeconomic factors associated with poor medication adherence for patients with type 2 diabetes

Marie Ekenberg[1], Miriam Qvarnström[1], Anders Sundström[1], Mats Martinell[2], Björn Wettermark[1]

1 Department of Pharmacy, Faculty of Pharmacy, Uppsala University, Uppsala, Sweden.
marie.ekenberg@farmaci.uu.se.
2 Department of Public Health and Caring Sciences, Uppsala University, Uppsala, Sweden.

Supplementary Table S3 Patients in each socioeconomic group for the four measurements of medication adherence: initiation of treatment before 30 days (I_{30}), initiation of second dispensation before 150 days (I_{150}), persistence with treatment after 12 months (P_{12}), and persistence with treatment after 24 months (P_{24}). Persistence is calculated based on the total population prescribed medication, not the patients initiating at I_{30}.

Characteristics		Initiation (I_{30})	Initiation (I_{150})	Persistence (P_{12})	Persistence (P_{24})
	N=8515	N (%) [95% CI]	N (%) [95% CI]	N (%) [95% CI]	N (%) [95% CI]
**Age**					
18-49 years	1979	1774 (89.6) [88.3-91.0]	1238 (62.6) [60.4-64.7]	996 (50.3) [48.1-52.5]	872 (44.1) [41.9-46.3]
50-64 years	2863	2646 (92.4) [91.5-93.4]	2122 (74.1) [72.5-75.5]	2010 (70.2) [68.5-71.9]	1894 (66.2) [64.4-67.9]
65-79 years	3048	2880 (94.5) [93.7-95.3]	2294 (75.3) [73.7-76.8]	2164 (71.0) [69.4-72.6]	1969 (65.0) [62.9;66.3]
≥80 years	625	567 (90.7) [88.4-93.0]	428 (68.5) [64.8-72.1]	359 (57.4) [53.6-61.3]	304 (48.6) [44.7-52.6]
**Sex**					
**Men**					
Living alone	1646	1498 (91.0) [89.6-92.4]	1157 (70.3) [68.1-72.5]	1100 (66.8) [64.6-69.1]	983 (59.7) [57.4-62.1]
Married/ Cohabiting	3053	2856 (93.5) [92.7-94.4]	2256 (73.9) [72.3-75.5]	2158 (70.7) [69.1-72.3]	2015 (66.0) [64.3-67.7]
**Women**					
Living alone	1509	1362 (90.3) [88.8-91.8]	1068 (70.8) [68.5-73.1]	917 (60.8) [58.3-63.2]	825 (54.7) [52.2-57.2]
Married/ Cohabiting	2307	2151 (93.2) [92.3-94.3]	1601 (69.4) [67.5-71.3]	1354 (58.3) [56.7-60.7]	1216 (52.7) [50.7-54.7]
**Country of birth**					
Sweden	6599	6191 (93.8) [93.2-94.4]	4867 (73.8) [72.7-74.8]	4428 (67.1) [66.0-68.2]	4043 (61.3) [60.1-62.4]
Other European countries	750	684 (91.2) [89.2-93.2]	518 (69.1) [65.8-72.4]	466 (62.1) [58.7-65.6]	426 (56.8) [53.3-60.3]
Rest of the World	1166	992 (85.1) [83.0-87.1]	697 (59.8) [57.0-62.6]	635 (54.5) [51.6-57.3]	570 (48.9) [46.0-51.8]
**Educational level**					
Primary	2246	2084(92.8) [91.7-93.8]	1637 (72.9) [71.0-74.7]	1511(67.3) [65.3-69.2]	1362 (60.6) [58.6-62.7]
Secondary	3781	3542 (93.7) [92.9-94.5]	2720 (71.9) [70.5-73.4]	2494 (66.0) [64.5-67.5]	2278 (60.2) [58.7-61.8]
University	2381	2173 (91.3) [90.1-92.4]	1669 (70.1) [68.3-71.9]	1480 (62.2) [60.2-64.1]	1362 (57.2) [55.2-59.2]
Missing	107	68 (63.6) [54.4-72.7]	56 (52.3) [42.9-61.8]	44 (41.1) [31.8-50.4]	37 (34.6) [25.6-43.6]
**Occupation**					
Employed	3802	3487 (91.7) [90.8-92.6]	2675 (70.4) [68.9-71.8]	2374 (62.4) [60.9-64.0]	2178 (57.3) [55.7-58.9]
Retired	4179	3932 (94.1) [93.4-94.8]	3092 (74.0) [72.7-75.3]	2873 (68.7) [67.3-70.2]	2600 (62.2) [60.7-63.7]
Long-term unemployment	154	124 (80.5) [74.3-86.8]	90 (58.4) [50.7-66.2]	77 (50.0) [42.1-57.9]	71 (46.1) [38.2-54.0]
Other unemployment	380	324 (85.3) [81.7-88.8]	225 (59.2) [54.3-64.2]	205 (53.9) [48.9-59.0]	190 (50.0) [45.0-55.0]
**Income**					
1st quartile	2128	1871 (88.1) [86.5-89.3]	1376 (64.7) [62.6-66.7]	1203 (56.5) [54.4-58.6]	1063 (50.0) [47.8-52.1]
2nd quartile	2127	1991 (93.6) [92.6-94.6]	1490 (70.1) [68.1-72.0]	1347 (63.3) [61.3-65.4]	1206 (56.7) [54.6-58.8]
3rd quartile	2131	2015 (94.6) [94.6-95.5]	1607 (75.4) [73.6-77.2]	1472 (69.1) [67.1-71.0]	1337 (62.7) [60.7-64.8]
4th quartile	2129	1990 (93.5) [92.4-94.5]	1609 (75.6) [73.8-77.4]	1507 (70.8) [68.9-72.7]	1433 (67.3) [65.3-69.3]
