# Supplementary material for: Socioeconomic factors associated with poor medication adherence in patients with type 2 diabetes
Source: Eur J Clin Pharmacol. 2023 Oct 23;80(1):53–63. doi: 10.1007/s00228-023-03571-8 (PMC10781833; doi:10.1007/s00228-023-03571-8)
Supplement: Supplementary file 4 — Supplementary file4 (PDF 120 KB) [file 228_2023_3571_MOESM4_ESM.pdf]

Socioeconomic factors associated with poor medication adherence for patients with type 2 diabetes

Marie Ekenberg[1], Miriam Qvarnström[1], Anders Sundström[1], Mats Martinell[2], Björn Wettermark[1]

1 Department of Pharmacy, Faculty of Pharmacy, Uppsala University, Uppsala, Sweden. marie.ekenberg@farmaci.uu.se.

2 Department of Public Health and Caring Sciences, Uppsala University, Uppsala, Sweden.

Supplementary Figure S4 Subgroups combining several socioeconomic factors associated with low initiation or persistence and the four measurements of medication adherence: initiation of treatment before 30 days (I_{30}), initiation of second dispensation before 150 days (I_{150}), persistence with treatment after 12 months (P_{12}), and persistence with treatment after 24 months (P_{24}). Persistence in this figure is calculated based on the total population prescribed medication, not the patients initiating at I_{30}.

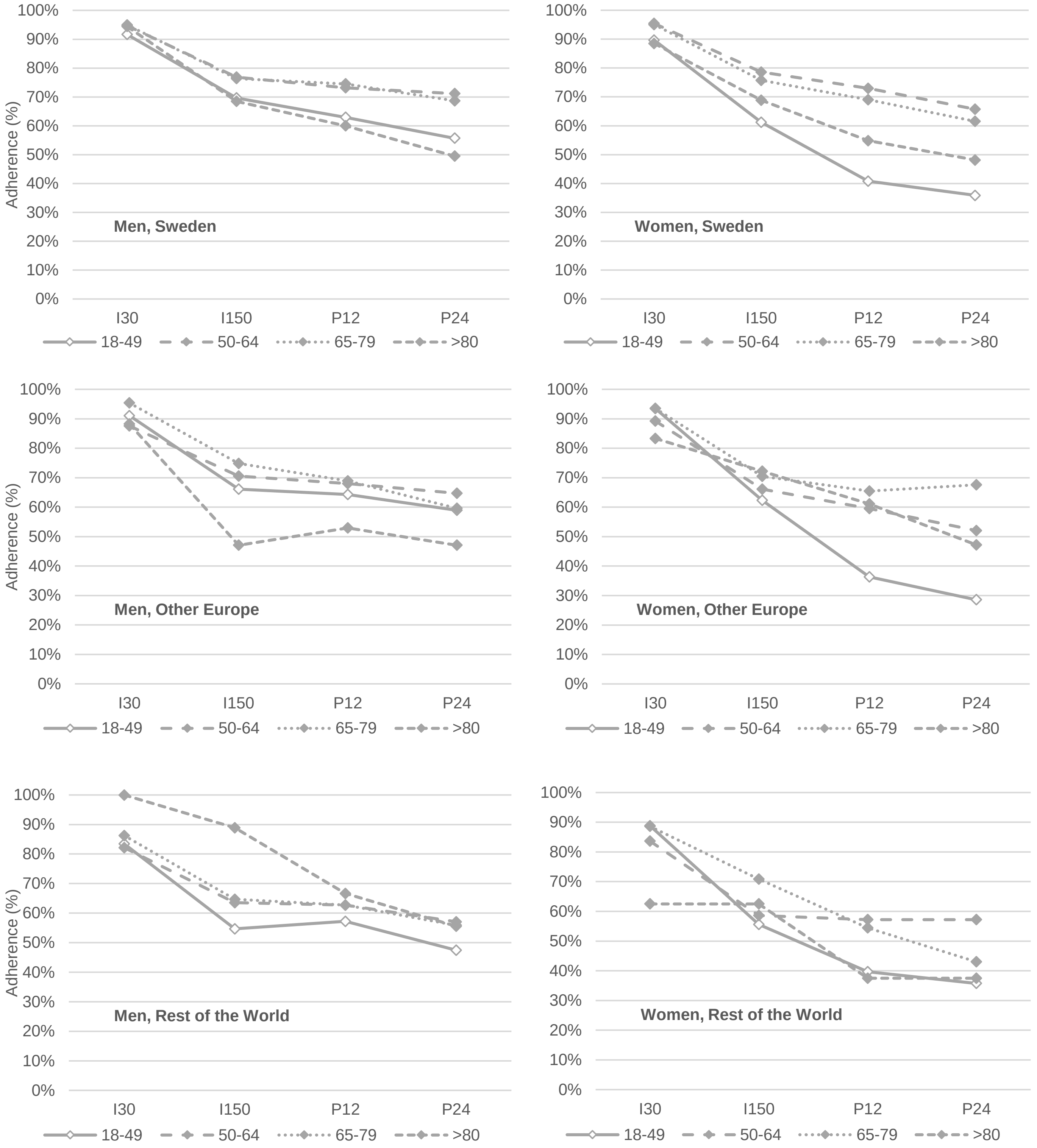

Figure S4.1 Initiation and peristence for pateints in the different age groups stratified on sex and birth country.

Table S4.1 Percentages and 95% confidence intervals for figure S4.1.

**Sex, country**	**Age**	**I30**	**I150**	**P12**	**P24**
Men, Sweden	18-49	91.6% (89.5-93.8)	69.6% (66.1-73.2)	62.9% (59.2-66.7)	55.7% (51.8-59.5)
	50-64	94.7% (93.5-95.9)	76.9% (74.5-79.2)	73.2% (70.8-75.6)	71.1% (68.7-73.6)
	65-79	94.9% (93.8-96.0)	76.4% (74.2-78.5)	74.5% (72.3-76.7)	68.7% (66.3-71.0)
	>80	94.4% (91.7-97.1)	68.4% (63.0-73.8)	60.0% (54.3-65.7)	49.5% (43.7-55.3)
Men, Other Europe	18-49	91.1% (83.6-92.8)	66.1% (53.7-78.5)	64.3% (51.7-76.8)	58.9% (46.0-71.8)
	50-64	87.6% (82.4-92.8)	70.6% (63.4-77.8)	68.0% (60.6-75.4)	64.7% (57.1-72.3)
	65-79	95.4% (92.0-98.7)	74.8% (67.9-81.8)	68.9% (61.5-76.3)	59.6% (51.8-67.4)
	>80	88.2% (72.9-103.6)	47.1% (23.3-70.8)	52.9% (29.2-76.7)	47.1% (23.3-70.8)
Men, Rest of the World	18-49	83.5% (78.7-88.2)	54.7% (48.3-61.0)	57.2% (50.9-63.5)	47.5% (41.1-53.8)
	50-64	82.2% (77.4-87.0)	63.6% (57.6-69.6)	62.8% (56.7-68.8)	57.1% (50.9-63.3)
	65-79	86.3% (79.6-93.0)	64.7% (55.4-74.0)	62.7% (53.4-72.1)	55.9% (46.2-65.5)
	>80	100.0% (100.0-100.0)	88.9% (68.4-109.4)	66.7% (35.9-97.5)	55.6% (23.1-88.0)
Women, Sweden	18-49	89.7% (87.4-92.0)	61.2% (57.6-64.8)	40.8% (37.2-44.5)	35.9% (32.3-39.5)
	50-64	95.4% (94.0-96.8)	78.6% (75.8-81.3)	73.0% (70.0-76.0)	65.7% (62.5-68.9)
	65-79	95.1% (93.8-96.4)	75.7% (73.1-78.3)	69.0% (66.2-71.8)	61.5% (58.6-64.5)
	>80	88.5% (84.7-92.3)	68.9% (63.4-74.4)	54.8% (48.9-60.8)	48.1% (42.2-54.1)
Women, Other Europe	18-49	93.5% (88.0-99.0)	62.3% (51.5-73.2)	36.4% (25.6-47.1)	28.6% (18.5-38.7)
	50-64	89.3% (83.7-94.8)	66.1% (57.7-74.5)	59.5% (50.8-68.3)	52.1% (43.2-61.0)
	65-79	93.5% (89.4-97.6)	70.5% (62.9-78.1)	65.5% (57.6-73.4)	67.6% (59.8-75.4)
	>80	83.3% (71.2-95.5)	72.2% (57.6-86.9)	61.1% (45.2-77.0)	47.2% (30.9-63.5)
Women, Rest of the World	18-49	88.8% (85.1-92.5)	55.6% (49.7-61.4)	39.7% (33.9-45.5)	35.7% (30.1-41.4)
	50-64	83.7% (78.6-88.7)	58.7% (52.0-65.3)	57.2% (50.5-63.9)	57.2% (50.5-63.9)
	65-79	88.6% (81.6-95.6)	70.9% (60.9-80.9)	54.4% (43.3-65.4)	43.0% (32.1-54.0)
	>80	62.5% (29.0-96.0)	62.5% (29.0-96.0)	37.5% (4.0-71.0)	37.5% (4.0-71.0)

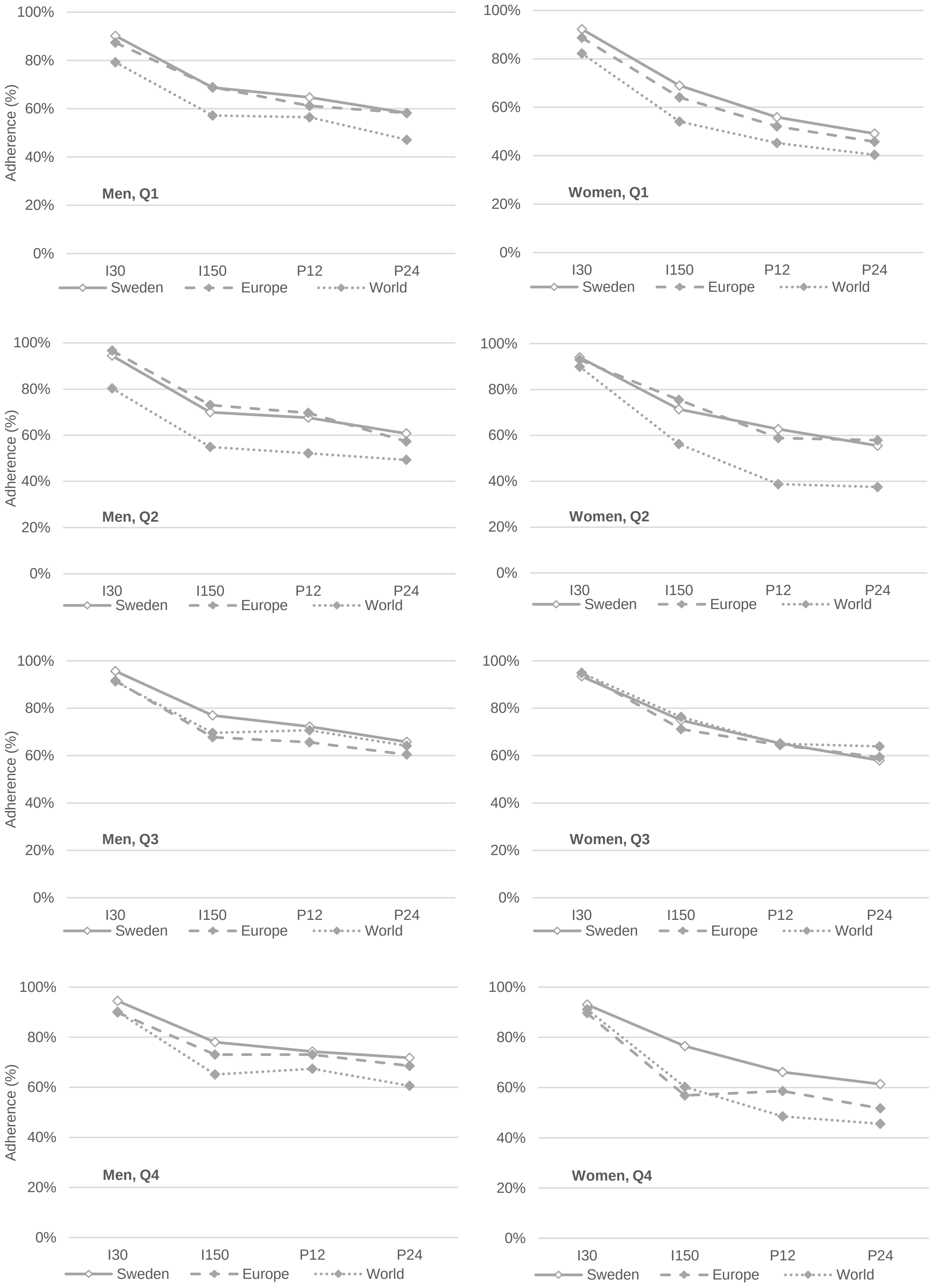

*Q1=1st income quartile, Q2=2nd income quartile, Q3= 3rd income quartile, Q4=4th income quartile.

Figure S4.2. Initiation and peristence for pateints from different birth country stratified on sex and income.

Table S4.2 Percentages and 95% confidence intervals for figure S4.2.

**Sex, income**	**Country**	**I30**	**I150**	**P12**	**P24**
Men, Q1	Sweden	90.2% (87.7-92.8)	68.8% (64.8-72.7)	64.8% (60.7-68.8)	58.2% (54.0-62.5)
	Europe	87.4% (81.0-93.8)	68.9% (60.0-77.9)	61.2% (51.8-70.6)	58.3% (48.7-67.8)
	World	79.3% (74.7-83.9)	57.2% (51.6-62.8)	56.5% (50.9-62.1)	47.2% (41.5-52.8)
Women, Q1	Sweden	92.2% (90.3-94.2)	69.0% (65.6-72.3)	55.9% (52.3-59.5)	49.1% (45.5-52.7)
	Europe	88.7% (83.5-93.9)	64.1% (56.2-72.0)	52.1% (43.9-60.3)	45.8% (37.6-54.0)
	World	82.3% (78.1-86.4)	54.1% (48.7-59.5)	45.3% (39.9-50.7)	40.4% (35.0-45.7)
Men, Q2	Sweden	94.4% (92.8-96.0)	70.0% (66.7-73.2)	67.6% (64.3-70.9)	60.7% (57.3-64.2)
	Europe	96.6% (92.9-100.4)	73.0% (63.8-82.3)	69.7% (60.1-79.2)	57.3% (47.0-67.6)
	World	80.3% (71.0-89.5)	54.9% (43.4-66.5)	52.1% (40.5-63.7)	49.3% (37.7-60.9)
Women, Q2	Sweden	94.0% (92.6-95.5)	71.4% (68.6-74.2)	62.7% (59.8-65.7)	55.5% (52.4-58.6)
	Europe	93.0% (88.3-97.7)	75.4% (67.5-83.3)	58.8% (49.7-67.8)	57.9% (48.8-67.0)
	World	90.0% (83.4-96.6)	56.3% (45.4-67.1)	38.8% (28.1-49.4)	37.5% (26.9-48.1)
Men, Q3	Sweden	95.7% (94.5-96.9)	77.0% (74.5-79.4)	72.2% (69.6-74.9)	65.8% (63.0-68.6)
	Europe	91.7% (86.1-97.2)	67.7% (58.4-77.1)	65.6% (56.1-75.1)	60.4% (50.6-70.2)
	World	91.3% (85.5-97.1)	69.6% (60.2-79.0)	70.7% (61.3-80.0)	64.1% (54.3-73.9)
Women, Q3	Sweden	93.4% (91.6-95.3)	75.0% (71.7-78.2)	65.1% (61.5-68.7)	58.0% (54.2-61.7)
	Europe	94.9% (89.3-100.5)	71.2% (59.6-82.7)	64.4% (52.2-76.6)	59.3% (46.8-71.9)
	World	94.8% (90.4-99.2)	76.3% (67.8-84.8)	64.9% (55.5-74.4)	63.9% (54.4-73.5)
Men, Q4	Sweden	94.5% (93.2-95.7)	78.0% (75.8-80.3)	74.3% (71.9-76.6)	71.8% (69.3-74.2)
	Europe	89.9% (83.6-96.2)	73.0% (63.8-82.3)	73.0% (63.8-82.3)	68.5% (58.9-78.2)
	World	90.2% (85.1-95.2)	65.2% (57.0-73.3)	67.4% (59.4-75.4)	60.6% (52.3-68.9)
Women, Q4	Sweden	93.1% (90.7-95.4)	76.6% (72.7-80.4)	66.2% (61.8-70.5)	61.4% (56.9-65.8)
	Europe	89.7% (81.8-97.5)	56.9% (44.2-69.6)	58.6% (45.9-71.3)	51.7% (38.9-64.6)
	World	91.2% (84.4-97.9)	60.3% (48.7-71.9)	48.5% (36.7-60.4)	45.6% (33.8-57.4)
