# Supplementary material for: Socioeconomic factors associated with poor medication adherence in patients with type 2 diabetes
Source: Eur J Clin Pharmacol. 2023 Oct 23;80(1):53–63. doi: 10.1007/s00228-023-03571-8 (PMC10781833; doi:10.1007/s00228-023-03571-8)
Supplement: Supplementary file 5 — Supplementary file5 (PDF 235 KB) [file 228_2023_3571_MOESM5_ESM.pdf]

Socioeconomic factors associated with poor medication adherence for patients with type 2 diabetes

Marie Ekenberg[1], Miriam Qvarnström[1], Anders Sundström[1], Mats Martinell[2], Björn Wettermark[1]

1 Department of Pharmacy, Faculty of Pharmacy, Uppsala University, Uppsala, Sweden.
marie.ekenberg@farmaci.uu.se.

2 Department of Public Health and Caring Sciences, Uppsala University, Uppsala, Sweden.

Supplementary Table S5 Logistic regression analysis including the full model$^{\alpha}$, reduced model$^{\beta}$ and crude odds ratios (OR) with 95% confidence intervals for factors associated with initiation of treatment before 30 days (I_{30}), initiation of second dispensation before 150 days (I_{150}), persistence with treatment after 12 months (P_{12}), and persistence with treatment after 24 months (P_{24}). The first part of the table (Age – Income) is shown in Fig 4 in the article.

Characteristics	Initiation (I_{30})	Initiation (I_{150})	Persistence (P_{12})	Persistence (P_{24})
	n= 8515<br>*Crude OR (95% CI)*<br>***Reduced model$^{\beta}$ OR (95% CI)***<br>Full model$^{\alpha}$ OR (95% CI)	n=8515<br>*Crude OR (95% CI)*<br>***Reduced model$^{\beta}$ OR (95% CI)***<br>Full model$^{\alpha}$ OR (95% CI)	n=7867 (92.4%)<br>*Crude OR (95% CI)*<br>***Reduced model$^{\beta}$ OR (95% CI)***<br>Full model$^{\alpha}$ OR (95% CI)	n= 7867<br>*Crude OR (95% CI)*<br>***Reduced model$^{\beta}$ OR (95% CI)***<br>Full model$^{\alpha}$ OR (95% CI)
**Age**				
18-49 years	1 (ref.)	1 (ref.)	1 (ref.)	1 (ref.)
50-64 years	*1.41 (1.15-1.72)*<br>***1.07 (0.85-1.33)***<br>0.95 (0.76-1.19)	*1.71 (1.51-1.94)*<br>***1.41 (1.22-1.61)****<br>1.32 (1.14-1.52)*	*2.47 (2.17-2.81)*<br>***1.89 (1.63-2.19)****<br>1.80 (1.55-2.09)*	*2.61 (2.30-2.96)*<br>***1.94 (1.69-2.24)****<br>1.84 (1.59-2.13)*
65-79 years	*1.98 (1.60-2.45)*<br>***1.36 (1.04-1.77)***<br>0.82 (0.53-1.25)	*1.82 (1.61-2.06)*<br>***1.45 (1.24-1.69)****<br>1.35 (1.05-1.72)	*2.36 (2.08-2.68)*<br>***1.76 (1.50-2.07)****<br>1.80 (1.38-2.34)*	*2.24 (1.98-2.53)*<br>***1.51 (1.29-1.77)****<br>1.51 (1.17-1.95)*
≥80 years	*1.12 (0.84-1.55)*<br>***0.88 (0.60-1.31)***<br>0.58 (0.34-0.98)	*1.30 (1.07-1.58)*<br>***1.09 (0.86-1.39)***<br>1.03 (0.75-1.41)	*1.35 (1.11-1.64)*<br>***1.20 (0.94-1.54)***<br>1.25 (0.90-1.73)	*1.20 (0.99-1.45)*<br>***1.00 (0.79-1.28)***<br>1.03 (0.75-1.41)
**Sex**				
**Men**				
Living alone	1 (ref.)	1 (ref.)	1 (ref.)	1 (ref.)
Married/Cohabiting	*1.43 (1.15-1.79)*<br>***1.29 (1.02-1.62)***<br>1.24 (0.98-1.57)	*1.20 (1.05-1.37)*<br>***1.08 (0.94-1.25)***<br>1.05 (0.91-1.21)	*1.12 (0.97-1.29)*<br>***0.98 (0.84-1.15)***<br>0.96 (0.82-1.12)	*1.26 (1.10-1.43)*<br>***1.11 (0.96-1.29)***<br>1.08 (0.93-1.25)
**Women**				
Living alone	*0.92 (0.72-1.16)*<br>***0.88 (0.68-1.13)***<br>0.98 (0.76-1.27)	*1.02 (0.88-1.19)*<br>***0.98 (0.83-1.15)***<br>1.05 (0.89-1.24)	*0.75 (0.63-0.88)*<br>***0.71 (0.60-0.85)****<br>0.75 (0.63-0.89)*	*0.80 (0.69-0.94)*<br>***0.81 (0.69-0.96)***<br>0.87 (0.73-1.03)
Married/Cohabiting	*1.36 (1.08-1.72)*<br>***1.39 (1.08-1.77)****<br>1.45 (1.13-1.86)*	*0.96 (0.83-1.10)*<br>***0.96 (0.82-1.10)***<br>1.01 (0.87-1.17)	*0.61 (0.53-0.71)*<br>***0.60 (0.51-0.70)****<br>0.63 (0.54-0.74)*	*0.68 (0.59-0.78)*<br>***0.67 (0.57-0.77)****<br>0.71 (0.61-0.83)*
**Country of birth**				
Sweden	1 (ref.)	1 (ref.)	1 (ref.)	1 (ref.)
Other European countries	*0.68 (0.52-0.90)*<br>***0.70 (0.53-0.93)***<br>0.77 (0.58-1.02)	*0.79 (0.68-0.94)*<br>***0.80 (0.67-0.95)****<br>0.84 (0.70-0.99)	*0.85 (0.71-1.01)*<br>***0.81 (0.68-0.97)***<br>0.86 (0.72-1.04)	*0.88 (0.75-1.03)*<br>***0.83 (0.70-0.99)***<br>0.88 (0.74-1.06)
Rest of the World	*0.38 (0.31-0.46)*<br>***0.42 (0.34-0.51)****<br>0.56 (0.45-0.70)*	*0.53 (0.46-0.60)*<br>***0.58 (0.50-0.66)****<br>0.64 (0.55-0.74)*	*0.71 (0.62-0.82)*<br>***0.81 (0.70-0.95)***<br>0.87 (0.74-1.03)	*0.72 (0.62-0.82)*<br>***0.78 (0.67-0.91)****<br>0.83 (0.70-0.97)
**Educational level**				
Primary	1 (ref.)	1 (ref.)	1 (ref.)	1 (ref.)
Secondary	*1.15 (0.94-1.42)*<br>***1.19 (0.96-1.48)***<br>1.05 (0.84-1.31)	*0.95 (0.85-1.07)*<br>***0.97 (0.86-1.10)***<br>0.89 (0.78-1.01)	*0.90 (0.80-1.02)*<br>***0.93 (0.81-1.06)***<br>0.88 (0.77-1.01)	*0.96 (0.85-1.07)*<br>***0.96 (0.85-1.09)***<br>0.91 (0.80-1.03)

University	*0.81 (0.66-1.01)*<br>***0.87 (0.69-1.09)***<br>0.79 (0.62-1.00)	*0.87 (0.77-0.99)*<br>***0.94 (0.82-1.07)***<br>0.85 (0.73-0.98)	*0.81 (0.71-0.92)*<br>***0.89 (0.77-1.02)***<br>0.81 (0.70-0.94)*	*0.89 (0.79-1.01)*<br>***0.94 (0.82-1.08)***<br>0.85 (0.74-0.98)
Missing	*0.14 (0.09-0.21)*<br>***0.16 (0.10-0.26)****<br>0.23 (0.15-0.38)*	*0.41 (0.28-0.61)*<br>***0.51 (0.34-0.77)****<br>0.62 (0.40-0.95)	*0.70 (0.42-1.17)*<br>***0.93 (0.55-1.64)***<br>1.01 (0.59-1.79)	*0.63 (0.39-1.04)*<br>***0.83 (0.49-1.41)***<br>0.94 (0.55-1.62)
**Occupation**				
Employed	1 (ref.)	1 (ref.)	1 (ref.)	1 (ref.)
Retired	*1.44 (1.21-1.71)*<br>***1.79 (1.23-2.68)***<br>1.32 (0.92-1.92)	*1.20 (1.09-1.32)*<br>***1.03 (0.84-1.27)***<br>0.96 (0.78-1.18)	*1.27 (1.15-1.41)*<br>***0.98 (0.79-1.22)***<br>0.96 (0.79-1.21)	*1.17 (1.07-1.29)*<br>***1.01 (0.82-1.24)***<br>0.99 (0.80-1.23)
Long-term unemployment	*0.37 (0.25-0.57)*<br>***0.37 (0.24-0.59)****<br>0.50 (0.32-0.81)*	*0.59 (0.43-0.83)*<br>***0.59 (0.42-0.84)****<br>0.79 (0.55-1.12)	*0.77 (0.53-1.12)*<br>***0.70 (0.48-1.04)***<br>0.81 (0.55-1.22)	*0.81 (0.56-1.16)*<br>***0.74 (0.50-1.09)***<br>0.89 (0.60-1.33)
Other unemployment	*0.52 (0.39-0.72)*<br>***0.55 (0.41-0.77)****<br>0.65 (0.47-0.91)	*0.61 (0.49-0.76)*<br>***0.66 (0.53-0.83)****<br>0.77 (0.61-0.98)	*0.81 (0.64-1.03)*<br>***0.95 (0.74-1.23)***<br>1.03 (0.79-1.34)	*0.85 (0.68-1.07)*<br>***1.03 (0.80-1.33)***<br>1.17 (0.91-1.52)
**Income**				
1st quartile	1 (ref.)	1 (ref.)	1 (ref.)	1 (ref.)
2nd quartile	*2.01 (1.62-2.50)*<br>***1.74 (1.37-2.23)****<br>1.24 (0.96-1.60)	*1.28 (1.12-1.45)*<br>***1.08 (0.93-1.25)***<br>0.97 (0.83-1.13)	*1.16 (1.02-1.33)*<br>***0.92 (0.79-1.08)***<br>0.92 (0.79-1.08)	*1.17 (1.03-1.33)*<br>***0.98 (0.84-1.14)***<br>0.96 (0.82-1.13)
3rd quartile	*2.39 (1.90-3.01)*<br>***2.07 (1.63-2.65)****<br>1.50 (1.16-1.95)*	*1.68 (1.47-1.91)*<br>***1.49 (1.29-1.73)****<br>1.33 (1.15-1.55)*	*1.51 (1.31-1.73)*<br>***1.27 (1.09-1.48)****<br>1.21 (1.03-1.42)	*1.50 (1.32-1.71)*<br>***1.28 (1.11-1.48)****<br>1.19 (1.02-1.39)
4th quartile	*1.97 (1.59-2.45)*<br>***1.72 (1.37-2.16)****<br>1.26 (0.97-1.63)	*1.69 (1.48-1.93)*<br>***1.49 (1.29-1.72)****<br>1.31 (1.12-1.54)*	*1.73 (1.51-1.99)*<br>***1.40 (1.20-1.62)****<br>1.29 (1.09-1.53)*	*1.96 (1.71-2.24)*<br>***1.55 (1.34-1.80)****<br>1.40 (1.19-1.65)*
**GFR**				
CKD1	1 (ref.)	1 (ref.)	1 (ref.)	1 (ref.)
CKD2	*1.71 (1.37-2.13)*<br>1.24 (0.96-1.60)	*1.43 (1.80-2.23)*<br>1.01 (0.87-1.18)	*1.58 (1.38-1.81)*<br>0.98 (0.84-1.16)	*1.70 (1.49-1.93)*<br>1.12 (0.96-1.31)
CKD3a	*1.39 (0.99-1.98)*<br>1.17 (0.79-1.78)	*1.36 (1.11-1.68)*<br>1.07 (0.84-1.18)	*1.09 (0.88-1.34)*<br>0.78 (0.61-1.01)	*1.12 (0.91-1.36)*<br>0.93 (0.73-1.18)
CKD3b	*1.57 (0.89-3.05)*<br>1.73 (0.92-3.51)	*1.17 (0.84-1.64)*<br>1.11 (0.77-1.63)	*0.82 (0.59-1.14)*<br>0.78 (0.53-1.14)	*0.61 (0.45-0.84)*<br>0.74 (0.51-1.07)
CKD4, CKD5	*0.74 (0.42-1.42)*<br>1.24 (0.65-2.50)	*0.62 (0.41-0.94)*<br>0.93 (0.58-1.47)	*0.35 (0.23-0.55)*<br>0.51 (0.31-0.84)	*0.30 (0.19-0.48)*<br>0.62 (0.36-1.03)
Missing	*1.22 (0.99-1.52)*<br>1.11 (0.85-1.47)	*1.22 (1.07-1.40)*<br>1.11 (0.94-1.33)	*1.07 (0.93-1.23)*<br>0.81 (0.67-0.97)	*1.22 (1.06-1.39)*<br>1.01 (0.85-1.21)
**HbA1c**				
≤48 mmol/mol	1 (ref.)	1 (ref.)	1 (ref.)	1 (ref.)
49-69 mmol/mol	*1.31 (1.06-1.62)*<br>1.26 (1.00-1.58)	*1.47 (1.30-1.66)*<br>1.41 (1.24-1.61)	*1.91 (1.68-2.17)*<br>1.65 (1.44-1.90)	*1.81 (1.60-2.04)*<br>1.55 (1.36-1.76)
≥70 mmol/mol	*1.17 (0.92-1.51)*<br>2.23 (1.67-2.98)	*1.15 (1.00-1.33)*<br>2.03 (1.72-2.41)	*1.22 (1.06-1.40)*<br>1.81 (1.52-2.16)	*1.23 (1.08-1.42)*<br>1.81 (1.53-2.14)
Missing	*0.65 (0.52-0.81)*<br>0.93 (0.73-1.19)	*0.83 (0.72-0.95)*<br>1.08 (0.93-1.26)	*0.91 (0.79-1.05)*<br>1.18 (1.01-1.39)	*0.89 (0.77-1.01)*<br>1.05 (0.90-1.23)
**Comorbidities**$^{\gamma}$				
Hypertension	*1.52 (1.29-1.79)*<br>1.12 (0.92-1.37)	*1.42 (1.29-1.56)*<br>1.11 (0.99-1.24)	*1.59 (1.44-1.76)*<br>1.21 (1.07-1.36)	*1.58 (1.44-1.73)*<br>1.27 (1.14-1.42)
Cardiovascular$^{\delta}$	*1.00 (0.80-1.27)*<br>0.83 (0.64-1.09)	*1.11 (0.96-1.27)*<br>1.00 (0.85-1.17)	*0.93 (0.81-1.07)*<br>0.75 (0.64-0.89)	*0.86 (0.75-0.98)*<br>0.74 (0.64-0.86)
Depression	*0.87 (0.65-1.21)*<br>1.11 (0.89-1.40)	*0.89 (0.74-1.08)*<br>1.10 (0.96-1.25)	*0.95 (0.84-1.07)*<br>1.07 (0.93-1.22)	*0.81 (0.72-0.91)*<br>0.92 (0.81-1.05)
Obesity	*0.99 (0.78-1.29)*<br>1.05 (0.80-1.41)	*1.18 (1.01-1.37)*<br>1.18 (1.00-1.40)	*0.92 (0.79-1.07)*<br>1.04 (0.88-1.24)	*0.70 (0.61-0.81)*<br>0.83 (0.70-0.97)
**Prescribed treatment**				
Metformin monotherapy	1 (ref.)	1 (ref.)	1 (ref.)	1 (ref.)
Insulins	*0.30 (0.25-0.38)*<br>0.30 (0.23-0.39)	*0.24 (0.20-0.28)*<br>0.24 (0.21-0.29)	*0.16 (0.13-0.19)*<br>0.17 (0.14-0.21)	*0.15 (0.12-0.17)*<br>0.16 (0.13-0.20)
Other monotherapy	*0.48 (0.37-0.62)*<br>0.50 (0.38-0.67)	*0.85 (0.71-1.02)*<br>0.92 (0.76-1.12)	*0.57 (0.48-0.68)*<br>0.73 (0.60-0.89)	*0.35 (0.29-0.41)*<br>0.48 (0.40-0.58)
Polytherapy	*0.25 (0.19-0.32)*<br>0.23 (0.17-0.31)	*0.29 (0.24-0.35)*<br>0.24 (0.20-0.30)	*0.35 (0.28-0.43)*<br>0.29 (0.23-0.36)	*0.31 (0.25-0.38)*<br>0.26 (0.21-0.33)

* Multivariable models remaining significant when using an 99% confidence interval.
[α]The full model included all other variables in the figure (Socioeconomic factors) and age, prescribed treatment (metformin monotherapy, insulins, other antidiabetic monotherapy, and polytherapy), CKD level, HbA1c, hypertension, cardiovascular disease, depression, obesity and year of prescription.
[β]The reduced models included age, prescribed treatment (metformin monotherapy, insulins, other antidiabetic monotherapy, and polytherapy), CKD level, HbA1c, hypertension, cardiovascular disease, depression, obesity and year of prescription.
[γ]Comparison group is not to have the diagnosis.
[δ]Cardiovascular disease groups include ischemic heart diseases (I20-I25), peripheral vascular disease (I70-I79), stroke/ TIA (I63, G45.9) and heart failure (I50)).
