# Supplementary material for: Socioeconomic factors associated with poor medication adherence in patients with type 2 diabetes
Source: Eur J Clin Pharmacol. 2023 Oct 23;80(1):53–63. doi: 10.1007/s00228-023-03571-8 (PMC10781833; doi:10.1007/s00228-023-03571-8)
Supplement: Supplementary file 6 — Supplementary file6 (PDF 116 KB) [file 228_2023_3571_MOESM6_ESM.pdf]

Socioeconomic factors associated with poor medication adherence for patients with type 2 diabetes

Marie Ekenberg[1], Miriam Qvarnström[1], Anders Sundström[1], Mats Martinell[2], Björn Wettermark[1]

1 Department of Pharmacy, Faculty of Pharmacy, Uppsala University, Uppsala, Sweden. *marie.ekenberg@farmaci.uu.se*.

2 Department of Public Health and Caring Sciences, Uppsala University, Uppsala, Sweden.

Supplementary Table S6 Sensitivity analysis comparing persistence to the initially prescribed antidiabetic medications and persistence to any antidiabetic therapy for both 12 ± 3 months and 24 ± 3 months

Characteristics	Persistence to initially prescribed medications (P_{12})	Persistence to any antidiabetic therapy	Persistence to initially prescribed medications (P_{24})	Persistence to any antidiabetic therapy
	**12 months ± 3 months**	**12 months ± 3 months**	**24 months ± 3 months**	**24 months ± 3 months**
**Sociodemographic data**	**n=7867 (92.4%) adjusted$^{\alpha}$ OR (95% CI)**	**n= 7867 adjusted$^{\alpha}$ OR (95% CI)**	**n= 7867 adjusted$^{\alpha}$ OR (95% CI)**	**n= 7867 adjusted$^{\alpha}$ OR (95% CI)**
**Age**				
18-49 years	1 (ref.)	1 (ref.)	1 (ref.)	1 (ref.)
50-64 years	1.80 (1.55-2.09)*	1.94 (1.63-2.32)*	1.84 (1.59-2.13)*	1.97 (1.66-2.34)*
65-79 years	1.80 (1.38-2.34)*	2.23 (1.65-3.01)*	1.51 (1.17-1.95)*	1.71 (1.27-2.30)*
≥80 years	1.25 (0.90-1.73)	1.39 (0.96-2.02)	1.03 (0.75-1.41)	0.94 (0.65-1.35)
**Sex**				
**Men**				
Living alone	1 (ref.)	1 (ref.)	1 (ref.)	1 (ref.)
Married/Cohabiting	0.96 (0.82-1.12)	0.94 (0.78-1.13)	1.08 (0.93-1.25)	0.98 (0.82-1.18)
**Women**				
Living alone	0.75 (0.63-0.89)*	0.69 (0.56-0.84)*	0.87 (0.73-1.03)	0.76 (0.62-0.92)
Married/Cohabiting	0.63 (0.54-0.74)*	0.66 (0.55-0.80)*	0.71 (0.61-0.83)*	0.71 (0.59-0.85)*
**Country of birth**				
Sweden	1 (ref.)	1 (ref.)	1 (ref.)	1 (ref.)
Other European countries	0.86 (0.72-1.04)	0.83 (0.67-1.03)	0.88 (0.74-1.06)	0.88 (0.72-1.09)
Rest of the World	0.87 (0.74-1.03)	0.70 (0.58-0.84)*	0.83 (0.70-0.97)*	0.66 (0.56-0.80)*
**Educational level**				
Primary	1 (ref.)	1 (ref.)	1 (ref.)	1 (ref.)
Secondary	0.88 (0.77-1.01)	0.92 (0.79-1.08)	0.91 (0.80-1.03)	0.93 (0.80-1.09)
University	0.81 (0.70-0.94)*	0.84 (0.71-1.00)	0.85 (0.74-0.98)*	0.86 (0.73-1.02)
Missing	1.01 (0.59-1.79)	1.69 (0.85-3.69)	0.94 (0.55-1.62)	1.20 (0.66-2.31)
**Occupation**				
Employed	1 (ref.)	1 (ref.)	1 (ref.)	1 (ref.)
Retired	0.96 (0.79-1.21)	0.73 (0.57-0.94)*	0.99 (0.80-1.23)	0.83 (0.65-1.07)
Long-term unemployment	0.81 (0.55-1.22)	0.80 (0.51-1.28)	0.89 (0.60-1.33)	1.06 (0.67-1.71)
Any unemployment	1.03 (0.79-1.34)	1.01 (0.75-1.37)	1.17 (0.91-1.52)	1.16 (0.87-1.57)
**Income**				
1st quartile	1 (ref.)	1 (ref.)	1 (ref.)	1 (ref.)
2nd quartile	0.92 (0.79-1.08)	0.87 (0.73-1.05)	0.96 (0.82-1.13)	0.98 (0.82-1.17)
3rd quartile	1.21 (1.03-1.42)*	1.11 (0.92-1.34)	1.19 (1.02-1.39)*	1.21 (1.01-1.44)*
4th quartile	1.29 (1.09-1.53)*	1.09 (0.89-1.32)	1.40 (1.19-1.65)*	1.30 (1.08-1.58)

* adjusted values, $p < 0.05$

$^{\alpha}$Values were adjusted for all other variables in the figure and additionally, prescribed treatment (metformin monotherapy, insulins, other antidiabetic monotherapy, and polytherapy), CKD level, HbA1c, hypertension, cardiovascular disease, depression, obesity and year of prescription.
